# Supplementary material for: Vesicant infusates are not associated with ultrasound-guided peripheral intravenous catheter failure: A secondary analysis of existing data
Source: PLoS One. 2022 Jan 27;17(1):e0262793. doi: 10.1371/journal.pone.0262793 (PMC8794136; doi:10.1371/journal.pone.0262793)
Supplement: S1 Appendix — (DOCX) [file pone.0262793.s001.docx]

| Appendix 1. List of medication use in study | | | |
| --- | --- | --- | --- |
| Amiodarone |  | Metoprolol | Compazine |
| Contrast |  | Metronidazole | Methylprednisolone Sodium Succinate |
| Dextrose 50% |  | Tazobactam | Lorazepam |
| Phenylephrine |  | Ketorolac | Diltiazem |
| Sodium Bicarbonate |  | Ondansetron | Diltiazem in Dextrose |
| Vancomycin |  | Haloperidol | Hydralazine |
| Calcium Gluconate |  | Fentanyl | Potassium chloride |
| Calcium Gluconate in Dextrose |  | Insulin | Hydrocortisone |
| Promethazine |  | Pantoprazole | Lidocaine |
| Promethazine in NaCl |  | Pantoprazole in NaCl | Metoclopramide |
|  |  | Morphine | Aztreonam |
|  |  | Ciprofloxacin | Prochlorperazine |
|  |  | Hydromorphone | Magnesium |
|  |  | Ceftriaxone | Azithromycin |
|  |  | Magnesium sulfate | Glycopyrrolate |
|  |  | Naloxone | Neostigmine |
|  |  | Propofol | Gentamicin in NaCl |
|  |  | Nitroglycerin in Dextrose | Thiamine |
|  |  | Midazolam | Meropenem |
|  |  | Furosemide | Dextrose in NaCl |
|  |  | Famotidine | Magnesium sulfate in Dextrose |
|  |  | Diphenhydramine | Sodium Acetate in Dextrose |
|  |  | Clindamycin Phosphate | Dextrose in Lactated Ringers |
|  |  | Clindamycin Phosphate in NaCl | Insulin Regular Human |
|  |  | Cefepime | Ferric Gluconate in NaCl |
|  |  | Prochlorperazine | Succinylcholine |
|  |  | Lactated Ringers | Ampicillin Sulbactam in NaCl |
|  |  | Heparin | Tazobactam in D5W |
|  |  | Heparin in Dextrose | Dexamethasone |
|  |  | Linezolid |  |
