# Supplementary material for: Vesicant infusates are not associated with ultrasound-guided peripheral intravenous catheter failure: A secondary analysis of existing data
Source: PLoS One. 2022 Jan 27;17(1):e0262793. doi: 10.1371/journal.pone.0262793 (PMC8794136; doi:10.1371/journal.pone.0262793)
Supplement: S2 Appendix — (DOCX) [file pone.0262793.s002.docx]

| Appendix 2. Effects of variables on multivariable Cox regression models | | | | | | |
| --- | --- | --- | --- | --- | --- | --- |
| Effects^¶^ |  | Model^§,⁑^  (without interaction) | |  | Model^‡,⁑^  (with interaction) | |
|  |  | Log-HR, β  (SE) | *p* value |  | Log-HR, β  (SE) | *p* value |
| Vesicant/Irritant Infusates  (yes *vs* no) |  | 0.158  (0.223) | 0.477 |  | -0.043  (0.282) | 0.878 |
| Ultrasound-guided PIVC  (UL *vs* SL) |  | -0.789  (0.235) | 0.001 |  | -1.016  (0.307) | 0.001 |
| Vesicant/Irritant Infusates  ×  Ultrasound-guided PIVC |  |  |  |  | 0.502  (0.433) | 0.246 |
| Age |  | 0.008  (0.007) | 0.248 |  | 0.008  (0.007) | 0.243 |
| Sex  (female *vs* male) |  | 0.114  (0.259) | 0.660 |  | 0.107  (0.259) | 0.679 |
| BMI |  | 0.008  (0.012) | 0.505 |  | 0.008  (0.012) | 0.499 |
| ESRD  (yes *vs* no) |  | 0.005  (0.303) | 0.987 |  | 0.014  (0.303) | 0.964 |
| SBP |  | -0.003  (0.004) | 0.515 |  | -0.003  (0.004) | 0.534 |
| Pulse rate |  | 0.005  (0.006) | 0.394 |  | 0.005  (0.006) | 0.399 |
| Depth of vein |  | 0.914  (0.348) | 0.009 |  | 0.943  (0.351) | 0.007 |
| *PIVC*, peripheral intravenous catheter; *UL*, ultra long; S*L*, standard long; *BMI*, body mass index; *ESRD*, end-stage renal disease; *SBP*, systolic blood pressure; *HR*, hazard ratio; *SE,* standard error.  ^¶^ Use of vesicant/irritant infusates was time-dependent variable.  ^§^ Results of regression model without the interaction term of vesicant/irritant infusates and ultrasound-guided PIVC were shown.  ^‡^ Results of regression model with the interaction term of vesicant/irritant infusates and ultrasound-guided PIVC were shown.  ^⁑^ Rounding errors of β and SE result in the different p-values shown on the same β and SE in both models. | | | | | | |
