# Supplementary material for: Vesicant infusates are not associated with ultrasound-guided peripheral intravenous catheter failure: A secondary analysis of existing data
Source: PLoS One. 2022 Jan 27;17(1):e0262793. doi: 10.1371/journal.pone.0262793 (PMC8794136; doi:10.1371/journal.pone.0262793)
Supplement: S3 Appendix — (DOCX) [file pone.0262793.s003.docx]

| Appendix 3. Causes of intravenous line removal by vesicants and irritant infusates and PIVC type | | | | | | |
| --- | --- | --- | --- | --- | --- | --- |
|  |  |  |  | Vesicant/Irritant Infusates | | |
| Causes of IV Removal |  | Ultrasound-guided PIVC |  | Yes |  | No |
| Phlebitis |  | UL |  | 0/41 |  | 3/90 |
|  |  | SL |  | 7/55 |  | 4/71 |
| Infiltration |  | UL |  | 2/41 |  | 4/90 |
|  |  | SL |  | 8/55 |  | 8/71 |
| Dislodgement |  | UL |  | 4/41 |  | 4/90 |
|  |  | SL |  | 0/55 |  | 3/71 |
| Occlusion |  | UL |  | 4/41 |  | 4/90 |
|  |  | SL |  | 2/55 |  | 3/71 |
| Leaking |  | UL |  | 3/41 |  | 2/90 |
|  |  | SL |  | 3/55 |  | 3/71 |
| Other |  | UL |  | 0/41 |  | 1/90 |
|  |  | SL |  | 1/55 |  | 2/71 |
| Unknown |  | UL |  | 5/41 |  | 5/90 |
|  |  | SL |  | 5/55 |  | 4/71 |
| *IV*, intravenous; *PIVC*, peripheral intravenous catheter; *UL*, ultra long; S*L*, standard long. | | | | | | |
