# Supplementary material for: Vesicant infusates are not associated with ultrasound-guided peripheral intravenous catheter failure: A secondary analysis of existing data
Source: PLoS One. 2022 Jan 27;17(1):e0262793. doi: 10.1371/journal.pone.0262793 (PMC8794136; doi:10.1371/journal.pone.0262793)
Supplement: Attachment — Submitted filename: Vesicant Manuscript_Author Response Letter.11.6.2021.AB.docx [file pone.0262793.s004.docx]

Dear Editor and Reviewers:

Thank you for your comments and edits to our submission. We are very excited that you are still considering our manuscript for publication. Below is a point by point response to each comment.

Editor Comment:

1. Please ensure that your manuscript meets PLOS ONE's style requirements, including those for file naming. The PLOS ONE style templates can be found at

https://journals.plos.org/plosone/s/file?id=wjVg/PLOSOne_formatting_sample_main_body.pdf and [https://journals.plos.org/plosone/s/file?id=ba62/PLOSOne_formatting_sample_title_authors_affiliations.pdf](https://secure-web.cisco.com/1B2eMvR0M0O4z1d4dRNuBmgNBNd28q0YaMUujBuBLYJXJu7ymIQMfub-YpWSPH6Rpzcq3QLmNemalaNxwF4vNCccJmFrhByAaZXe8Wz_N2Lfs842FIyQS9nIMYCKb4ilmj9nFvP0SmiIF7n_yYQczHdAwz-mJ-G7I5EOOuV1CK3lPuDG-bZy6vzfNIgcVAOMw141p6aprvCrPS9MSzmWUkH_eBC5yD2renmxDnSQNFCTqPhpRznIZhjekTE45jwBeEUKqpfyUFqe5KrAlizMHEnGfGcF_7uY8-Z_bqZ2WPyNeoWSIgE-55IGbaigXFEzKVTZgMxTRa4cmmkF0wgD5tA/https%3A%2F%2Fjournals.plos.org%2Fplosone%2Fs%2Ffile%3Fid%3Dba62%2FPLOSOne_formatting_sample_title_authors_affiliations.pdf).

Author Response:

The title page has been modified to meet the PLOS ONE style requirement.

Editor Comment:

2. Thank you for stating the following in the Competing Interests/Financial Disclosure*  section:

“I have read the journal's policy and the authors of this manuscript have the following competing interests: AB is a paid consultant for B. Braun. He provides expertise regarding vascular access products and services. All other authors have no relevant disclosures.”

We note that one or more of the authors are employed by a commercial company: B. Braun

Author Response:

AB is not employed by B. Braun. AB is a key opinion leader and has provided expertise in vascular access to B. Braun as a paid consultant.

Editor Comment:

a. Please provide an amended Funding Statement declaring this commercial affiliation, as well as a statement regarding the Role of Funders in your study. If the funding organization did not play a role in the study design, data collection and analysis, decision to publish, or preparation of the manuscript and only provided financial support in the form of authors' salaries and/or research materials, please review your statements relating to the author contributions, and ensure you have specifically and accurately indicated the role(s) that these authors had in your study. You can update author roles in the Author Contributions section of the online submission form.

Please also include the following statement within your amended Funding Statement.

“The funder provided support in the form of salaries for authors [insert relevant initials], but did not have any additional role in the study design, data collection and analysis, decision to publish, or preparation of the manuscript. The specific roles of these authors are articulated in the ‘author contributions’ section.”

If your commercial affiliation did play a role in your study, please state and explain this role within your updated Funding Statement.

Author Response:

Thank you for the inquiry. The funding statement has been amended; however somewhat differing from the suggestion. The funder did NOT provide a salary to AB. There is no employer-employee relationship. B. Braun provided consulting fees to AB for work unrelated to this study. The funding statement has been updated to clarify that AB has received monies for consulting work that are unrelated to this publication.

The new funding statement is:

“AB is a paid consultant for B.Braun. He provides expertise regarding vascular access products and services. The funder did not provide any support for this manuscript. All other authors have no relevant disclosures.”

Editor Comment:

b. Please also provide an updated Competing Interests Statement declaring this commercial affiliation along with any other relevant declarations relating to employment, consultancy, patents, products in development, or marketed products, etc.

Within your Competing Interests Statement, please confirm that this commercial affiliation does not alter your adherence to all PLOS ONE policies on sharing data and materials by including the following statement: "This does not alter our adherence to  PLOS ONE policies on sharing data and materials.” (as detailed online in our guide for authors http://journals.plos.org/plosone/s/competing-interests) . If this adherence statement is not accurate and  there are restrictions on sharing of data and/or materials, please state these. Please note that we cannot proceed with consideration of your article until this information has been declared.

Please include both an updated Funding Statement and Competing Interests Statement in your cover letter. We will change the online submission form on your behalf.

Author Response:

The Competing Interests Statement has been updated. Both funding and competing interests statements are now also included in the cover letter.

Editor Comment:

3. Please include a caption for figure 1.

Author Response:

A caption was added in the text.

Editor Comment:

4. Please include your tables as part of your main manuscript and remove the individual files. Please note that supplementary tables (should remain/ be uploaded) as separate "supporting information" files.

Author Response:

Done.

Editor Comment:

5. Please include captions for your Supporting Information files at the end of your manuscript, and update any in-text citations to match accordingly. Please see our Supporting Information guidelines for more information: [http://journals.plos.org/plosone/s/supporting-information.](http://secure-web.cisco.com/19dMjDEAbI8X3X6yUL69pyMlDj5fux6Ol7bxtKKW-xPd4GweMzDAURwQ-f_Wd7HHF54fJIc_ynpxuhND5jEea5n14AP7r-UbVBDdq77nCBT2rHYsYakGZP_RQPTqUP2ycnkQkW7jlTHW0TgWwL29Y-C3eedXpc98Y7G2JsMVZebn-YGp_cJ7-_Oj4qr5sxOCPUAqVBU39Q0YSrY1C3dHnchVORN8jd8YXRpJjwD3-yXcQ2CBCWrA5GnBUiKYla5yiJls65hPX392FEl9MZKZXwsaAEpxBDAeIeiKTYq7XmmvO_TuVNRPZRqiuTmkUqIkZWHqpOOrtyOgdcBpqpjoGfQ/http%3A%2F%2Fjournals.plos.org%2Fplosone%2Fs%2Fsupporting-information)

Author Response:

Done.

Editor Comment:

6. Please review your reference list to ensure that it is complete and correct. If you have cited papers that have been retracted, please include the rationale for doing so in the manuscript text, or remove these references and replace them with relevant current references. Any changes to the reference list should be mentioned in the rebuttal letter that accompanies your revised manuscript. If you need to cite a retracted article, indicate the article’s retracted status in the References list and also include a citation and full reference for the retraction notice.

Author Response:

Done.

**Reviewer #1: PONE-D-21-15885: statistical review**

Reviewer Comment:
SUMMARY. This is a secondary analysis of an existing randomized control trial. It estimates the influence of vesicant infusates on peripheral intravenous catheters (PIVC) failure. The core statistical analysis relies on a Cox regression model where time up to PIVC failure is the dependent variable and use of vesicant infusates is the principal time-varying covariate. Although the results are sound and the statistical methods seem generally correct, I list below some points that need clarification.

Author Response:

Thank you for your review. We will do our best to address your comments.

Reviewer Comment:
1. I didn’t find any information about the censored cases. How many censored cases do we have for each catheter type? Can we assume that censoring is not informative?

Author Response:

In this secondary analysis, our main independent variable is the status of infusates use. In Figure 1 of this manuscript, the number at risk at each time point (days) was listed for “status of infusates use”; for vesicant use, 44 (failure)/34 (censored) and for no vesicant use, 50 (failure)/129 (censored). As for cases of censoring and failure for “catheter type” in the original RCT design, it was shown in Bahl et al. (2020) [reference: #16]. For SL catheter, 41 (failure)/90 (censored); for UL catheter, 53 (failure)/73 (censored). We assume the censoring is non-informative; the index IV removal not related to failure could be due to the completion of the IV use or discharge. When the index IV failure before the completion of therapy or discharge, rescue IV were subsequently used until the completion of therapy or discharge. Censoring times of the subjects are not influenced by times of failure.

Reviewer Comment:
2. Table 1 displays a significant interaction between catheter type and use of infusates (p=0.041) that has not been discussed. How do the authors interpret this interaction?

Author Response:

In this study, we were mainly exploring the potential impact of infusates use on IV failures through a secondary analysis of a RCT data. The original data was collected based on the randomization of catheter type rather than the randomization of infusates use. Also, the use of infusates in Table 1 was defined as “any” use of infusates until the index IV removal rather than observation at baseline. This significant association (p=0.041) of catheter type and use of infusates might be the bias of non-randomization on infusates use. It reveals there is potential imbalance of catheter type by use of infusates in Table 1. Hence, when exploring the effect of infusates use on the risk of IV failure, it was also adjusting for catheter type in multivariable regression models.

Reviewer Comment:
3. The material relating to the Cox regression results is not fully clear. I’d welcome two tables of results, which respectively display both the Cox regression with all covariates plus the interaction term of vesicant infusates and catheter type, and the Cox regression with all the covariates but without the interaction term. It is important to see whether the covariate effects change significantly when the interaction effect is removed.

Author Response:

Thank you for comment. For this request, we provided more results as supplemental materials on **Appendix 2**. This is a secondary analysis of a RCT data and the interaction term of vesicant infusates and catheter type on IV failures is NOT statistically significant. Hence, as we mentioned in analysis section, we mainly discussed the adjusted effect of vesicant infusates on the risk of IV failures in regression model *without* interaction term. As such, we keep the original Table 2 shown in the text. In the text (analysis section) and the footnote of Table 2, we further indicated that additional information can be found on **Appendix 2.**

On **Appendix 2**, the regression coefficients of log-HR were shown to see how it changed with/without interaction term in models. As we know, in a regression model with the interaction term, an additive effect (i.e., regression coefficient of the interaction term) was estimated. Conceptually, the shown effects (regression coefficients) of vesicant infusates and catheter type are NOT directly comparable in regression models with/without the interaction term, respectively. More precisely, when estimating (/interpreting) effect of vesicant infusates in a regression model with the interaction term, it should be conditioning on each specific catheter type. *[see below] In terms of the information shown on appendix 2, it further indicated that the adjusted effect (aHR) of vesicant infusates were not statistically significant increased risk of IV failure, conditioning on catheter type.*

| Cox regression Model **with interaction term** | | |
| --- | --- | --- |
| Effect | aHR  (95% CI) | P value |
| Vesicant/Irritant Infusates (yes *vs* no)  at (conditioning on) catheter type = SL | 0.96  (0.55 to 1.66) | 0.878 |
| Vesicant/Irritant Infusates (yes *vs* no)  at (conditioning on) catheter type = UL | 1.58  (0.82 to 3.07) | 0.175 |

So, in manuscript, it is adequate when the conclusion/discussion was derived from regression models *without* the interaction term along with the adjusted effect (aHR:1.17, p-value=0.477) of vesicant infusates on IV failures across catheter types (shown on Table 2).

Reviewer Comment:

4. Cox regression models are capable of handling time-varying covariates by organizing the data as counting processes (i.e. multiple records for each subject that reflect the change of status). I don’t fully understand why the preliminary analysis that involves the Simon-Makuch method and the Mantel-Byar test is really needed here. Please clarify. In particular, I suspect that Mantel-Byar test is equivalent to the ordinary log-rank test when the data are arranged as counting processes…

Author Response:

Thank you for comment. We agree your viewpoint. In analysis, we also tried to generate the unadjusted Kaplan-Meier curves to explore the potential median survival time on a time-varying vesicant infusates before we carried out univariable and multivariable Cox regression analyses. As such, we mentioned the Simon-Makuch on the modified Kaplan-Meier curves. In the text, we retain that we explored the modified K-M curves when data was arranged as counting process. In figure, we replace the annotated ‘Mantel-Byar’ by ‘log-rank’.

**Reviewer #2:**

Reviewer Comment:

Congratulations on your excellent study and clearly written manuscript. This subject area is one of great interest. While I appreciated the results reflecting extravasation incidence and non-serious outcomes, I would have liked to see a bit more data and explanation on the reasons for PIVC failure within the groups.

Author Response:

Thank you for your review Dr. Moureau! We have added a supplemental table (Appendix 3) to include etiology of failure. This cannot be added to the main text as we are restricted in space and table limits based on journal requirements.

**Reviewer #3:**

Reviewer Comment:

The authors should be congratulated, it is a well written, prepared, and presented paper, however I do have some comments.

Author Response:

Thank you for your review. We will do our best to address your comments in our revision.

Reviewer Comment:
Title: "Vesicant infusates are not associated with ultrasound-guided peripheral intravenous catheter failure: A time-dependent extended analysis of a randomized controlled trial" not sure what time-dependent extended analysis means, perhaps say 'secondary analysis'. Using failure as outcome should imply a time-to-event analysis. It may not be appropriate to mention RCT in the title, since this study was not randomised on vesicant/non-vesicant drugs. It's a secondary analysis of a dataset that happens to come from an RCT.

Author Response:

The study title has been modified to: “**Vesicant infusates are not associated with ultrasound-guided peripheral intravenous catheter failure: A secondary analysis of existing data”**

Reviewer Comment:
"Further, the healthcare costs associated with complications are astounding and the financial burden of premature catheter failure is at a minimum of $1.5 billion nationally.7,8" Perhaps also mention the impact on the patient.

Author Response:

We agree and have added the following sentence: “PIVC failure leads to significant patient harm in the form of repeated insertion attempts, treatment delays, venous depletion, prolongation of hospital stay, psychological and physical stress from needlesticks, and increased rates of nosocomial infections.”

Reviewer Comment:
Need to mention the setting, e.g. name of hospital.

Author Response:

We added description of the study setting to the study sample section.

Reviewer Comment:
Need to specify the definition of failure, 5mL flushing was mentioned, but the determination of failure based on other signs/symptoms (e.g. pain) should be detailed. These are listed in the supplement but should be in the main text.

Author Response:

Thanks for this comment. The complications are now listed in the main text.

Reviewer Comment:
Over-fitting of the Cox model could be a concern at this sample size with this many co-variables, especially if the survival analysis is split. May not need to adjust for all these co-variables. Variable selection rationale should be detailed.

Author Response:

Thank you for comment. In this study, the survival analysis is not split. In analysis, the failure time was split into the format of counting process when analyzing the effect of time-varying vesicant infusates on IV failure. In addition, this is a secondary analysis of a RCT data [reference #: 16]. We adjusted for the same pre-specified covariates as reference #16 (Bahl et al., 2020) for the same outcome (dependent variable: IV failure) and mentioned it in the text. We further make it clear in the text.

During process of analysis, we also have considered a rule of thumb, “one in 10 rule” (i.e., 10 events per variable) that the relevant references were listed as follows. In this data, there are ~ 94 events of IV failure. It is adequately acceptable to include ~ 10 variables in regression model, except for the outcome (dependent variable).

[1] Concato J, Peduzzi P, Holfold TR, et al. Importance of events per independent variable in proportional hazards analysis. I. Background, goals, and general strategy. J Clin Epidemiol 1995; 48:1495–501.

[2] Peduzzi P, Concato J, Feinstein AR, et al. Importance of events per independent variable in proportional hazards regression analysis. II. Accuracy and precision of regression estimates. J Clin Epidemiol 1995; 48:1503–10.

[3] Vittinghoff E and McCulloch CE. Relaxing the rule of ten events per variable in logistic and Cox regression. J Clin Epidemiol 2007; 165:710–718.

Reviewer Comment:
KM plot: should be truncated at around 7 days (remaining data are practically meaningless)

Author Response:

Thank you for comment. It has been revised.

Reviewer Comment:

Table 1. What is 'line failure'? Is this the primary outcome? Should be moved to Table 3.

Author Response:

It is the primary outcome, the index IV failure. This descriptive information has been mentioned in the text and the frequencies (or proportions) of IV (line) failure can be inferred from Table 2. We have deleted it from Table 1. Because Table 3 is related to cox regression analysis, we feel that it may not be necessary to be repeat again in Table 3.

Reviewer Comment:

Table 2 and 3 should be swapped?

Author Response:

Thank you. We corrected errors on the label.

Reviewer Comment:

Table 2. What would be the result if the vesicant variable is treated as dichotomous (not time-dependent)? A simple Cox regression?

Author Response:

Herein, we provided results in Cox regression as follows. Vesicant Infusates was simply defined as any use rather than time-dependent feature as counting process until the index IV removal. It also demonstrates that there was no statistically significant increased risk of IV failure due to vesicant use.

| Effect | Univariable Cox regression | p value | Multivariable Cox regression | p value |
| --- | --- | --- | --- | --- |
|  | Unadjusted HR  (95% CI) |  | Adjusted HR  (95% CI) |  |
| Vesicant Infusates  (yes *vs* no) | 1.04  (0.69 to 1.58) | 0.852 | 0.95  (0.62 to 1.47) | 0.832 |

Reviewer Comment:

Cannot see the descriptive statistics of the number of vesicant doses (0/1/2/3).

Author Response:

We added this information into Table 1.

Reviewer Comment:
How do you explain that the effect of vesicant in Table 1 was significant but not significant in the advanced analyses? Should be in Discussion.

Author Response:

In Table 1, it only descriptively indicated the difference on the proportions (frequencies) of the index IV failure by the status of “any” vesicant use until the index IV removal (p=0.02). (1) In this study, we were mainly exploring the potential impact of infusates use on IV failures through a secondary analysis of a RCT data. The original data was collected based on the randomization of catheter type rather than the randomization of infusates use. There might exist the bias of non-randomization on infusates use resulting in this difference. (2) On the other hand, the effect of vesicant on the risk of (the index) IV failure is also involved with the insertion duration until the index IV removal due to complete/discharge (censored) or failure. Hence, when assessing the effect of vesicant in time-to-event Cox regression approach, *accounting for duration of insertion until IV removal*, it is much adequate to assess/explain the effect of vesicant use and the corresponding significance on the risk of IV failure. So, this may occur when applying the univariable Cox regression. (3) Moreover, it is not surprising that when further adjusting for variables with strong effect (e.g., catheter type) on IV failure, the effect of vesicant became much non-significant in multivariable regression. We believe that depending on the appropriateness of analytic approach applied to the design of data collection, it may occur. In analysis, we clearly mentioned what analytic approach was adopted.

The following sentence is in the discussion:

“While on the surface results indicate that any use of caustic infusates may increase the likelihood of PIVC failure, when time of administration was considered in the survival analysis, we determined that caustic infusates did not increase the risk of PIVC failure.”

Reviewer Comment:
Please check this research poster as it may be relevant: https://metronorth.health.qld.gov.au/hhps-2020/CLIN-0111.pdf

Author Response:

Thank you for this information. It is interesting. Since it is a poster, it is difficult to make any comment/comparison between their findings and results in our study. However, compared to our study data, there is significant difference that authors synthesize data from 7 RCTs and 2 cohort study. It mixes with experimental and observational designs as well as adults and pediatrics. From the viewpoint of analysis, we believe that in addition to age and sex, it may need to control for more covariates in multivariable Cox regression. Perhaps, the significance or strength of the adjusted effect might change. Alternatively, stratified Cox regression could be considered (e.g., the stratification factor: RCT/cohort study or adults/pediatrics) as well. It might have the influence on estimates of effects and the corresponding significance.
