# Supplementary material for: Vesicant infusates are not associated with ultrasound-guided peripheral intravenous catheter failure: A secondary analysis of existing data
Source: PLoS One. 2022 Jan 27;17(1):e0262793. doi: 10.1371/journal.pone.0262793 (PMC8794136; doi:10.1371/journal.pone.0262793)
Supplement: Attachment — Submitted filename: Author Response Letter PLOS 12.20.2021.docx [file pone.0262793.s005.docx]

Dear Dr. Miquel Vall-llosera Camps,

Thank you for your continued consideration of our submission PONE-D-21-15885R1 titled “Vesicant infusates are not associated with ultrasound-guided peripheral intravenous catheter failure: A secondary analysis of existing data”. Below is a point by point response to remaining concerns.

Best regards,

Amit Bahl

**Reviewer Comment:** One correction: the reference to INS Standards is not correct
13 Gorski LA. The 2016 infusion therapy standards of practice. Home Healthcare Now. 2017;35(1):10–8.
The correct reference:
Gorski L, Hadaway L, Hagle M, Broadhurst D, Clare S, Kleidon T, Meyer B, Nickel B, Rowley S, Sharpe E, Alexander M. (2021) Infusion Therapy Standards of Practice, 8th Edition. Journal of Infusion Nursing. 2021;44(1S):S1-S224. doi:10.1097/NAN.0000000000000396

**Author Response:** Thanks for catching this mistake. This reference has been revised.

**Reviewer Comment:**  In the Abstract/Conclusions, the first sentence should be in past tense and words "in this study" or similar should be added.

**Author Response:** The document has been modified to include the suggested verbiage.
